# An Emotional Bond: The Relationship between Maternal Stress and Offspring Disease

**DOI:** 10.1289/ehp.119-a488a

**Published:** 2011-11-01

**Authors:** Tanya Tillett

**Affiliations:** Tanya Tillett, MA, of Durham, NC, is a staff writer/editor for *EHP*. She has been on the *EHP* staff since 2000 and has represented the journal at national and international conferences.

Studies have suggested a potential association between maternal stress during pregnancy and an increased risk of specific diseases in offspring, prompting calls for a closer examination of stress as an environmental health factor. A new comprehensive study based on a large population-based cohort in Denmark explores this association further by examining maternal stress during pregnancy and the development of a wide spectrum of pediatric diseases [*EHP* 119(11):1647–1652; Tegethoff et al.].

The researchers examined prospective data for 66,203 mother–child pairs enrolled in the Danish National Birth Cohort. The mothers gave birth between 1996 and 2003, and the median child age at the end of followup was 6.2 years.

The researchers assessed two types of stress during each woman’s pregnancy—“life stress” and “emotional stress”—based on information reported by mothers at about 30 weeks’ gestation. Life stress refers to the mother’s perceived life burdens, e.g., work, housing, and human relations. Emotional stress refers to the mother’s feelings, e.g., irrational fear, hopelessness, and irritation. The researchers also collected data on disease diagnoses reported to the Danish National Hospital Register for the study children.

On average, mothers reported both life and emotional stress as being stronger before birth than after. The researchers observed associations between maternal life stress during pregnancy and a variety of childhood ailments during followup, including infectious and parasitic diseases, mental and behavioral disorders, and diseases of the eye, ear, skin, respiratory system, digestive system, musculoskeletal system, and genitourinary system. Maternal emotional stress during pregnancy was associated with an increased risk of infectious disease only—a finding that contradicts some previous studies. The authors write that the discrepancies may result from differences across studies in classifying emotional stress or disease outcomes.

**Figure:**
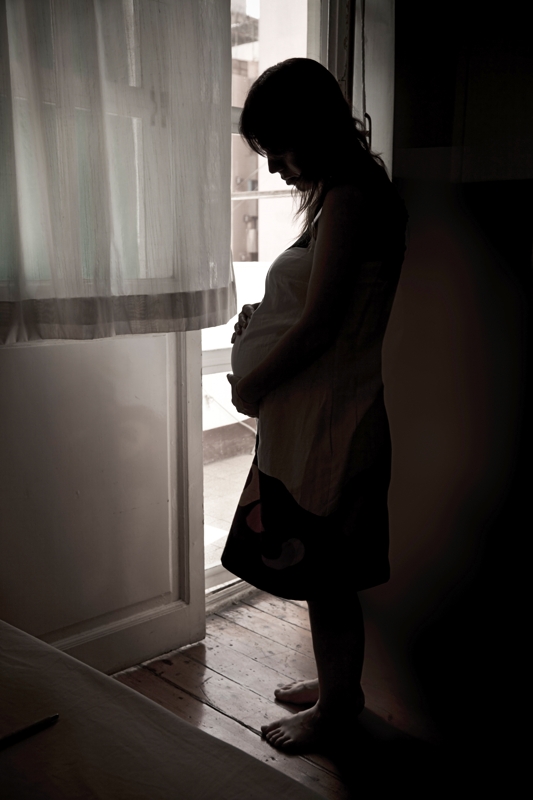
A growing body of literature backs up the folk wisdom of staying calm for the sake of the baby. © Ugurhan Betin/iStockphoto

Limitations to the study include a lack of data on the timing of mothers’ stress during pregnancy (each organ system has a specific critical window of intrauterine susceptibility) and the possibility of uncontrolled confounding factors such as chemical exposures. However, the findings overall corroborate and build upon those of earlier studies and underscore the need for strategies to reduce maternal stress during pregnancy. They write that future studies should look in more detail at offspring diseases related to maternal stress and should focus on determining the underlying mechanisms, which may help improve preventive approaches and interventions.

